# The lung, the niche, and the microbe: Exploring the lung microbiome in cancer and immunity

**DOI:** 10.3389/fimmu.2022.1094110

**Published:** 2023-01-17

**Authors:** Mai Huynh, Meredith J. Crane, Amanda M. Jamieson

**Affiliations:** Department of Molecular Microbiology & Immunology, Brown University, Providence, RI, United States

**Keywords:** lung microbiome, lung cancer, pulmonary immunity, tumor micreoenvironment (TME), tumor immunity, cancer therapy, lung microbiota

## Abstract

The lung is a complex and unique organ system whose biology is strongly influenced by environmental exposure, oxygen abundance, connection to extrapulmonary systems *via* a dense capillary network, and an array of immune cells that reside in the tissue at steady state. The lung also harbors a low biomass community of commensal microorganisms that are dynamic during both health and disease with the capacity to modulate regulatory immune responses during diseases such as cancer. Lung cancer is the third most common cancer worldwide with the highest mortality rate amongst cancers due to the difficulty of an early diagnosis. This review discusses the current body of work addressing the interactions between the lung microbiota and the immune system, and how these two components of the pulmonary system are linked to lung cancer development and outcomes. Bringing in lessons from broader studies examining the effects of the gut microbiota on cancer outcomes, we highlight many challenges and gaps in this nascent field.

## 1 Introduction

The human microbial milieu consists of a broad network of microorganisms across multiple organ systems that includes viruses, fungi, and, predominantly, bacteria (1). Each microbe localizes to a niche that suits their nutrient and oxygen preferences, where they regulate the local immunity and modulate the nutrient microenvironment (2). The lung’s unique access to oxygen and other airborne substances leads to microbiota that are highly distinct from other organ sites (3). The respiratory immune system necessitates an artillery of immune cells, whose functions are not only to identify and target infection and foreign substances, but also to control immunopathology during inflammation (4–6). This is particularly salient in diseases such as lung cancer, as cancer cells can manipulate host immunity to evade immune-initiated cell death, while inducing specialization of tumor-specific immune cells that can cater to tumor growth.

Lung cancer remains the third most prevalent cancer worldwide, with non-small cell lung carcinoma (NSCLC) accounting for 84% of those cancers and small cell lung carcinoma (SCLC) accounting for the other 16% (7). While efforts have been made to advance therapeutics in treating lung cancer, issues remain in identifying the cause of disease, either endogenous or exogenous, as well as modulating the robustness and specificity of the immune response. Due to the lung microbiota’s tight influence on pulmonary immunity, it is necessary to understand how the lung microbiota may not only potentiate disease during dysbiosis but may also be key in regulating the immune response during common cancer treatments. However, the sparseness of the lung microbiota has prevented sequencing strategies from capturing species-level determinations, making it difficult to describe the precise role of the lung microbiota in the context of disease (**Supplementary Figure 1**). Furthermore, most extractions are from sputum or bronchoalveolar lavage fluid (BALF), which are less invasive but indicate microbial compositions that are inconsistent with that of lobectomies (8, 9). Mouse models have allowed scientists to study whole-lung microbial extracts, but these studies fail to parallel human microbial dysbiosis (9). There are other limitations inherent to mouse models for microbiome research that present challenges. For example, the immune system of germ-free mice deviates significantly from healthy mice (10). While antibiotic-treated mice may better mimic natural immune responses, many studies do not acknowledge the differential effects antibiotic administration routes may have, making it difficult to disentangle local versus systemic disruptions to the microbiota and their resultant effects (11).

**Figure 1:**
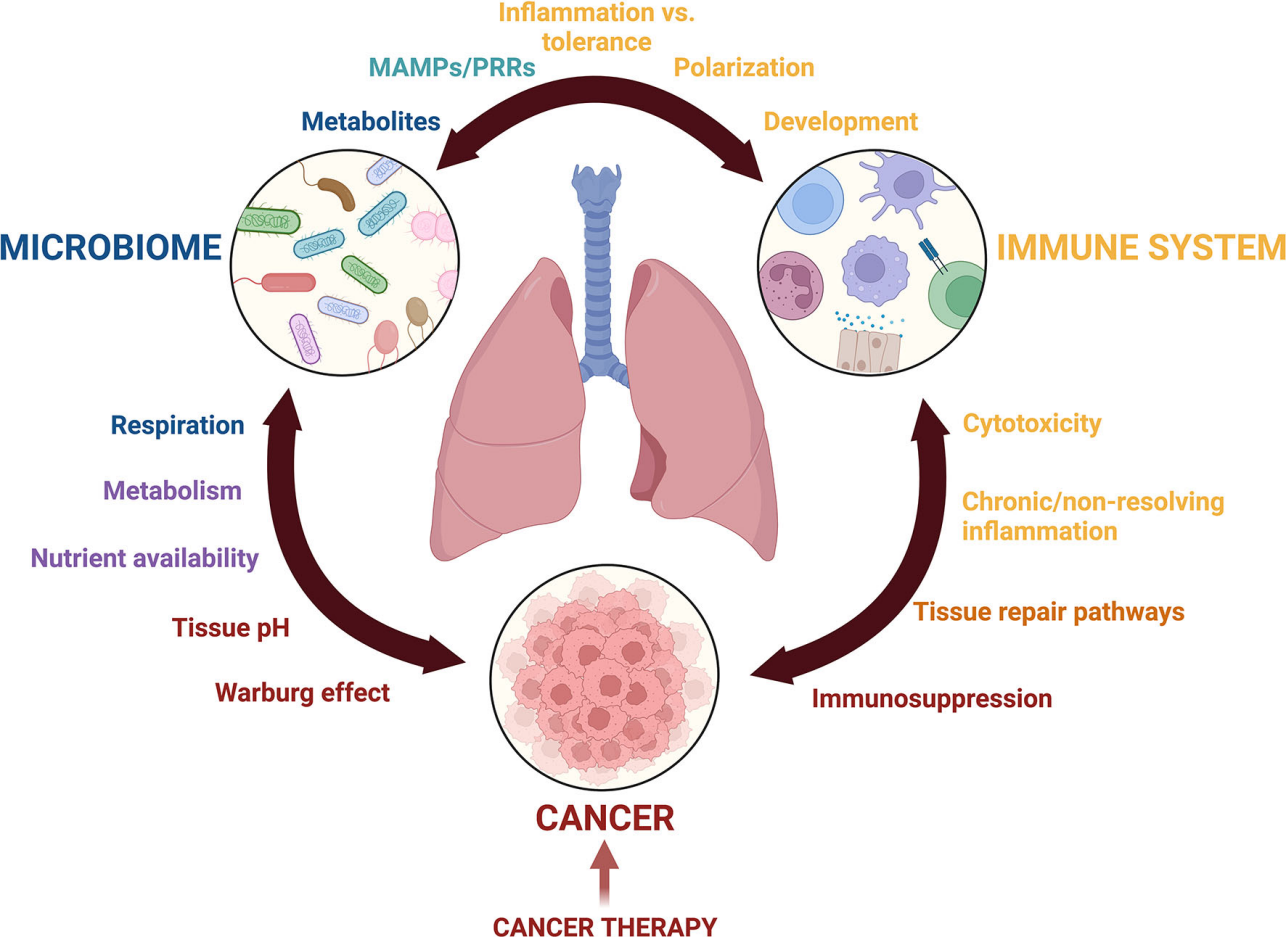
Crosstalk among the lung microbiota, pulmonary immune system, and the lung cancer microenvironment is complex. Communication mediated by metabolites, microbial associated molecular patterns (MAMPs), pattern recognition receptors (PRRs), inflammatory mediators, growth factors, and nutrient availability drives changes to Microbial diversity, inflammation, and potentially cancer development and progression. The balance of these interactions is further complicated by cancer treatments including immunotherapy, radiation, and chemotherapy. This figure was created with Biorender.com.

These barriers in lung microbiota research have been detrimental to the field of lung cancer, resulting in its research lagging behind other lung diseases or gut microbial research (12). Many recent opinion pieces and reviews have nicely discussed how the microbiome, in general, interacts with cancer. This narrative review focuses on the current state of lung resident microbiota characterization in healthy and cancerous lungs, and we present these findings in the context of the unique biology of the lung microenvironment and pulmonary immunity. Furthermore, we call upon discoveries from gut microbial research and disease in order to underscore the role of the microbiota in cancer and emphasize the importance of similarly researching the lung microbiota in lung cancer. The article highlights the potential for this field to provide insight into the development and progression of lung cancer as well as patient responsiveness to cancer treatments, while emphasizing the remaining gaps and unanswered questions that will be key in guiding future research (**Figure 1**).

## 2 Development of lung microbiota and pulmonary immunity

The human lung was previously believed to be sterile due to the inability of scientists to grow lung microbes outside of the lung in typical cultures, but with the advancement of sequencing technologies, scientists have discovered microbial communities that specifically occupy the lung (13, 14). The lungs are a complex barrier surface designed to facilitate gas exchange while providing protection against inhaled particulate matter and infectious organisms. The large surface area of the alveoli encompasses an extensive mucosal barrier that aids in the transfer of oxygen, making it ideal for the growth of microbes (15). The trachea, leading into the lung, is lined with cilia, which filter certain microbes and particulates. The oral cavity and its microbiota are constant sources of microbial exposure to the trachea and lung, and have been found to influence the microbes identified within the lung, although debate remains as to whether micro-aspirations of upper airway microbes are the primary source of the lung microbiota as opposed to direct introduction from external sources throughout life, as is the case with the gut (8, 12, 16). Major ecological determinants of the respiratory microbiota include inhalation and immigration of microbes from the oral and tracheal cavities as well as from the environment, regular immune surveillance and respiratory clearance, and the local growth conditions, including but not limited to pH, temperature, oxygen availability, nutrient availability, growth dominance of other organisms, and immune cell signaling (17, 18). This wide range of determinants results in constant, low-level turnover, suggesting time-contingent variance that complicates the study of the lung’s microbial system (3).

The lung microbiota primarily interact with the lung epithelium and alveolar macrophages (19). The lung epithelium can secrete antimicrobial peptides and mucus in response to the microbiota while alveolar macrophages serve as sentinels that can differentiate between pathogens versus symbionts (20, 21). The lung microbiota may influence the activation of these cells, especially as lung immunity is modulated by instances of chronic inflammation, such as during cancer. Because of its proximity to the lung microbiota, pulmonary immunity has evolved to respond to dysbiosis. For instance, commensal microbes harbor microbial associated molecular patterns (MAMPs) that canonically trigger downstream inflammation *via* activation of various PRRs, including NOD-like receptors, RIG-I-like receptors, C-type lectin receptors, and AIM2-like receptors that activate transcription factors NF-kB, MAPKs, and IRFs to drive pro-inflammatory signaling (22–24). Mouse models have shown that the presence of lung commensal bacteria decreased the severity of influenza and influenza-induced lung injury *via* the priming and differentiation of alveolar macrophages, indicating the importance of the lung microbiota in tempering the immune response during disease (25). However, there is emerging evidence that prolonged stimulation of PRRs by airway microbiota may result in suppressed innate immune responses, and hence innate immune tolerance (26). The precise mechanisms behind microbial-induced specialization remain unclear. However, most immune cells, both resident and infiltrating, undeniably act and differentiate in accordance to signals from the microbiota and express a combination of ligands as necessitated by either beneficial microbes or pathogens. This allows for a fine-tuned response to pathogens versus symbionts, allowing adaptability to changes in the lung microbiota (24).

## 3 Lung cancer and the role of the lung microbiota

The lung’s constant access to oxygen, its extended capillary network, and its interface with carcinogens in the environment can facilitate the spontaneous formation of tumors (27). Both NSCLC and SCLC typically begin their formation near or at the alveolus, and as the disease progresses, the cancer spreads outwards to the more peripheral regions of the lung as well as upwards into the trachea (28). SCLC is more tightly correlated with cigarette smoking than NSCLC, but both cancers are highly heterogeneous and difficult to diagnose early, leading to high rates of morbidity and mortality (29, 30). While many oncogenes have been identified as endogenous drivers of lung cancer, such as KRAS in NSCLC and RB in SCLC, the role of the microbiota in manipulating established pathways of oncogenesis is less understood (31). While a wide variety of bacterial, viral, and fungal microorganisms are associated with lung tumors, microorganisms causing direct oncogenesis in lung cancer have not been established (32). For instance, exposure to influenza was correlated with a 1.09-fold higher risk of lung cancer, although it is uncertain that this specific exposure caused oncogenesis. Other viruses that have been found in lung tumors include human papillomavirus (HPV), John Cunningham virus (JCV), Merkel cell polyomavirus (MCPyV), Epstein-Barr virus (EBV), and Jaagsiekte sheep retrovirus (JSRV), but more clinical studies are necessary to confirm their role in lung cancer (33, 34). Early work studying the role of infections in tumorigenesis found that mice exposed to a respiratory infection of *Mycoplasma pulmonis* had an increased incidence of lung neoplasms after exposure to carcinogens compared to germ-free mice and specific-pathogen-free (SPF) mice (35). Scientists have shown that airway epithelial cells exposed *in vitro* to the supernatant of *Veillonella*, a taxa associated with lung cancer, led to the upregulation of ERK, PI-3K and IL-17A pathways (36). In mice injected with the carcinogen NNK, the intranasal administration of lipopolysaccharide (LPS) resulted in an increased number and size of lung tumors, along with an upregulation of the pro-inflammatory and pro-proliferative transcription factors NF-kB and Akt (37).

While microbes and microbial products can directly or indirectly influence cancer development, the presence of cancer can likewise drive pulmonary dysbiosis (38). In the healthy, steady-state human lung, the most commonly appearing genera in 16S sequencing of BALF included (from most to least abundant) *Streptococcus, Prevotella* and *Veillonella*, which have been confirmed across other healthy BALF sequencing analyses (39, 40). These same genera, along with *Corynebacteria*, *Ralstonia*, and *Staphylococcus* were found in non-cancerous tissue or BALF in lung cancer patients, whereas in cancerous tissue or BALF, Firmicutes as a phylum remained the most abundant (although *Prevotella* becomes the most abundant genera, followed by *Bifidobacteirum*, *Acinetobacter*, and *Ruminococcus*); other abundant phyla in the tumor tissue included Bacteroides, as well as the genera *Actinomyces*, with *Rothia* more abundant in NSCLC patients compared to SCLC patients (13, 41–43). Some bacteria that have been identified using 16S sequencing as potential biomarkers due to their abundance in lung cancer samples include the phylum of Proteobacteria, as well as the genera *Veillonella, Capnocytophaga*, and *TM7-3* (44–46). In sputum extracts of a pilot study utilizing metagenomic sequencing, three bacteria, including *Granulicatella adiacens*, *Streptococcus intermedius*, and *Mycobacterium tuberculosis*, were statistically significantly more abundant in four lung cancer positive patients (47). However, many meta-analyses of the lung microbiota in the context of lung cancer have concluded that amongst compositional studies, the results are not consistent enough to identify specific genera or species as true biomarkers of disease (48). For instance, these studies indicated that Proteobacteria were identified in sputum and BALF samples, but were not found in the tissue samples themselves, indicating another issue with sampling of the lung microbiota (49). This makes it more difficult to identify microbial biomarkers of disease. Furthermore, healthy human lung sampling has primarily relied on BALF extractions or oral lavages, as tissue biopsies can only be studied when a patient requires lung surgical resection due to disease (such as during tumor removal) (50). Finally, the vast majority of compositional studies utilize 16S sequencing due to its accessibility and compatibility with lower microbial biomass samples, but unfortunately this method does not resolve the bacterial identification farther than the genus level (**Supplementary Figure 1**). Although there have been studies that utilized whole-shotgun metagenomic sequencing for species-level identification of the microbiota in lung cancer, the sample sizes were small, and more work is necessary to conclusively identify disease-associated microbes (51, 52). As more of these studies emerge, we will better be able to understand the composition and activity of the lung microbiota in the context of lung cancer.

Inflammation can drive cancer development, providing a potential link between immunomodulatory microbes and lung cancer. The presence of certain microbes, as well as persistent exposure to inhaled particulates or infections can lead to non-resolving inflammation in the lung, which can set the stage for neoplastic transition (53). Germ-free and antibiotic-treated mice were resistant to adenocarcinoma in a KRAS-p53 mouse model of NSCLC and the presence of microbiota in the lung was associated with MyD88 activation in myeloid cells. This led to activation of γδT cells *via* the production of IL-1β and IL-23, while IL-17 derived from activated γδT cells resulted in downstream inflammation (54). Bacteria can also drive inflammation through presentation of MAMPs, and certain MAMPs may be more or less immunogenic depending on their structure. For example, differences in LPS structure can make certain bacteria more immunostimulatory (55–58). Microbial products, such as short-chain fatty acids (SCFAs) have also been shown to be correlated with higher neutrophil counts in cystic fibrosis patients and may play a role in regulating inflammation in the lung (59, 60). Other bacterial metabolites, including spermidine and spermines, are protective in the lungs of asthmatic patients, promoting Src kinase and indoleamine 2,3-dioxygenase 1 (IDO1) activation while reducing NF-kB activation in dendritic cells (61, 62). While there are clear links between microbial products and immune modulation, much remains to be learned about how these interactions influence lung cancer development.

In addition to microbial-derived metabolites, cancer cells in the lung tumor tissue can dramatically alter the metabolic and immune landscape by excessively utilizing glucose and glutamine as energy sources *via* aerobic glycolysis, otherwise known as the Warburg effect (63). Furthermore, the relatively high pulmonary concentration of lactate provides another carbon source in NSCLC (64–67). The steady-state lung environment is unique in that it is glucose poor, which helps to restrict microbial growth, but this can be altered during states of prolonged inflammation (68). Such changes in the metabolic environment influence the function of immune cells (69). For example, CD8+ T cells do not acquire sufficient glucose in the presence of tumors in a mouse sarcoma model, which impairs T cell effector functions (70). In addition to nutrient competition, the overproduction and accumulation of tumor-derived lactate and lactic acid and hypoxia in the extracellular environment can cause T cell anergy and dampen the inflammatory functions of innate leukocytes (71). The balance between the regulatory functions of CD4+ T cells and cytotoxic capabilities of CD8+ T cells in response to tumors has been well studied. Prior studies have shown that the breadth of the T cell landscape in NSCLC is closely tied with tumor mutational burden, marked by an increased proportion of dysfunctional CD8+ and CD4+ T cell subsets (72). Furthermore, researchers have found that the KRAS mutation, commonly found in NSCLC tumors, increased the recruitment of Th17 cells, which was vital in generating and sustaining inflammation during the early stages of cancer (73). Previous work has shown that certain alterations of the lung microbiota can facilitate a similar sustained inflammation *via* activation of Th17 cells, suggesting that the lung microbiota may play a role in T cell mediated disease during lung cancer (74–76). Tumor cells also possess immunomodulatory properties, and will often downregulate MHC class I, escaping recognition by cytotoxic CD8+ T cells (77). However, the downregulation of MHC class I, along with the upregulation of stress ligands by cancer cells, will activate NK cells, inducing NK cell cytotoxic capabilities and production of inflammatory cytokines and chemokines (78–80). In a B16 melanoma model with metastasis to the lung, pulmonary microbiota depletion shifted the balance of regulatory T cells and cytotoxic leukocytes such that upon antibiotic treatment, regulatory T cells were depleted, leading to an increase in protective NK cells and activated T cells (81). These data indicate the important role that the lung microbiota may have in regulating the immune system in response to cancer.

Macrophages also play important roles in the tumor microenvironment. Interestingly, the tumor environment generally favors macrophage polarization away from classical activation and towards the pro-tumorigenic alternative activation state (82–84). In the lung this trend is complicated by the phenotypically and functionally diverse set of lung resident and recruited macrophages that have been characterized in cancerous tissue (85). For instance, tumor associated macrophages (TAMs) are immunosuppressive, and can contribute to metastasis (86). However, in antibiotic treated mice, alveolar macrophages were found to have elevated expression of CCL24, a chemokine that mediates cancer growth. The presence of commensal microbes was necessary to steady CCL24 expression at low levels to allow for an anti-tumor immune response (87). Information about TAM origin, differentiation, characteristics, how they differ from alveolar macrophages, and how they arrive at the lung tumor has yet to be determined (88, 89).

## 4 Lessons from the gut

While research of the lung microbiota continues to expand, the comprehensive gut microbiota research may provide clues as to microbial behavior in the context of human health and disease and underpin the significance of these microbes in tuning immune responses. However, we caution the extent to which conclusions are extrapolated from gut microbial research for several reasons. First, the gut and the lung are not only different in structure and function, but also harbor vastly different types of microbes. For instance, while the lung contains a mix of both aerobic and anaerobic bacteria, with greater aerobic bacteria during health, the gut contains predominantly anaerobic bacteria during health, with a shift towards aerobic bacteria during disease (90). Furthermore, gut immunity is centered around regulation in response to the multitude of microbiota therein, whereas lung immunity is primed to readily respond to viral or bacterial infections as well as foreign particulates (91, 92). However, we underline the significance of gut microbiome research in not only educating our understanding of microbial behavior and effect in human systems, but also in pointing out areas of necessary research in the lung microbiome field. In this section, we will focus on how gut microbial research can be understood in the context of the lung microbiota in lung cancer, rather than crosstalk between the two organs (otherwise known as the “gut-lung axis”), which has been extensively reviewed elsewhere (93–95).

The transition from chronic inflammation to malignant disease has been well-documented in colon carcinogenesis, and its mechanisms have been associated with the gut microbial milieu and its role in inducing a chronically inflamed state (96, 97). Individuals with inflammatory bowel diseases have an elevated risk for developing colon cancer, and many persistent infections are carcinogenic, such as *Helicobacter pylori*, a major risk factor for gastric cancer (98). Microbes that colonize mucosal surfaces display heterogeneity in their immunogenicity regarding antibody recognition, and *Helicobacter pylori* was among a group of interstitial microbes that were highly bound by IgA, suggesting that these species are targeted by specific immune responses and have the potential to drive intestinal inflammation (99). *Fusobacterium nucleatum* is another human gut microbe that has been correlated with colorectal cancer, and the presence of this microbe was associated with elevated TNF-a and NF-kB expression, as well as elevated expression of K-ras (100–102). Microbial products and metabolites, such as SCFAs, secondary bile acids, triethylamine, and arginine-derived polyamines, also possess immunomodulatory activities that have been assessed primarily in the context of non-pulmonary tissues (62, 103, 104). While the metabolome of the lung microbiota has not been well established in the context of lung cancer, previous work in the gut has implicated the use of metabolites as biomarkers of health and disease. In the gut microbiota of lung cancer patients versus healthy controls, it was found that butyric and pentanoic acids, aldehydes, ketones, terpenes, and *p*-cresol were associated with health, while metabolites like dodecane, 2,6-dimethyl-4 heptanone, and methyl isobutyl ketone were primarily expressed in the guts of lung cancer patients (105). Between responders and non-responders, it was found that the presence of SCFAs (such as propionic, butyric, acetic, and valeric acid), lysine, and nicotinic acid in the gut were associated with better response to checkpoint immunotherapies (105). Similarly, in clinical trials, it was found that 2-pentanone and decane were associated with early progression of NSCLC after treatment with PD-1 therapy, whereas SCFAs, lysine, and nicotinic acid were correlated with better response and long-term effects (106). Human metabolite studies have also shown that tryptophan catabolites have an anti-inflammatory effect in the gut and contribute to gut homeostasis, whereas butanal is associated with inflammation and cancer (107, 108). Together, these findings suggest that shifts in the abundance of certain microbes and their metabolites can influence the inflammatory response at mucosal sites and can be used to determine disease and response to treatments. Furthermore, this research emphasizes the need to explore similar indicators local to the lung tumor which may be a significant milestone in understanding the dynamics of lung cancer.

### 4.1 Cancer therapies

#### 4.1.1 Radiation therapy and chemotherapy

Little has been uncovered on the effect of radiation therapy (RTX) or chemotherapy on the microbiota. Mouse studies have shown that ionizing RTX can alter both the diversity and the abundance of the gut microbiota and thereby alter the efficacy of RTX or increase the irradiation associated injury; specifically, *Akkermansia* has been reported to be associated with irradiation injuries in the gut *via* mucosal degradation, increasing tissue susceptibility to injury, and potentially altering the ability of the mucosal layer to uptake both drugs and nutrients (109). Irradiated germ-free mice were also found to have fewer apoptotic cells in their intestinal mucosal linings, along with increased immunogenic cell death, and systemic inflammation, which may disrupt microbes therein (110). RTX has also been shown to affect the circadian rhythm of patients, which induces gut microbial dysbiosis, further feeding into the decline of patient circadian rhythm and affecting the success of the treatment, although the mechanisms of this dysbiosis require further research (110).

Similar to RTX, research is still necessary to understand the effect of chemotherapy on the microbiota, especially of the lung microbiota. The first-in-line treatments for many cancers include platinum-based therapies, or other agents that target DNA replication mechanisms, which reduce the integrity of cell division, although these agents tend to be non-specific, targeting both eukaryotic and prokaryotic cells (111). It was found that in both germ-free mice and mice whose gut microbiota were depleted by antibiotics, the efficacy of platinum-based chemotherapies was reduced in MC38 colon carcinomas, EL4 lymphomas, and B16 melanomas, all of which are normally susceptible to platinum-based therapies (112). In particular, the production of reactive oxygen species (ROS) by tumor-infiltrating hematopoietic cells was significantly reduced during gut dysbiosis, but after the introduction of the known probiotic *Lactobacillus acidophilus*, there is a recovery in some of the antitumor effects of cisplatin (112). Evidence has shown that probiotic gut bacteria, such as *Lactobacillus acidophilus* and *Bifidobacterium bifidum*, resulted in the inhibition of NOX proteins. This prevented early ROS production in the gut epithelia and activated TLR2 and TLR4, ultimately protecting the mucosal layer and preventing cytotoxic damage during chemotherapy (113–115). These results further suggest the significance of the microbiota in modulating the efficacy and toxicity of therapy. Together, this research suggests that RTX and chemotherapy can alter the microbiota composition by interfering with mucosal integrity and proper immune cell engagement, which in turn affects the success of these therapies. As such, it is of paramount importance that these therapies are further explored in the context of the lung microbiota to better understand how the microbial microenvironment of the tumor is not only affected by these therapies, but how they may play a role in the efficacy of these therapies.

#### 4.1.2 Immunotherapy

Due to the promise of immunotherapy as a treatment option, especially for patients who have had limited success with other types of treatment, it becomes important to better understand if the effect of immunotherapy on the microbiota may be involved in the experienced toxicity from immunotherapy. Despite showing promise in the reduction of metastatic disease and increased survival rate, immunotherapy has high rates of discontinuation among patients due to toxicity (116, 117). Common immunotherapies utilized for the treatment of cancer, such as CTLA-4, PD-1 and PD-L1 inhibitors, promote T cell antitumor activities, but also result in a distinct multi-organ inflammatory profile, which could in turn influence the microbiota (118, 119).

Research on the gut microbiota during cancer and immunotherapy have indicated that specific consortia of microbes are essential in influencing therapeutic response and specifically tempering the robust immune response associated with immunotherapy (120). For instance, antibiotic-treated mice with NSCLC showed a poorer response to immunotherapy compared to untreated mice; specifically, mice that were better responders to immunotherapy had an overrepresentation of *Alistipes shahii* in their gut microbiota, which correlated with increased TNF-α production by tumor-associated myeloid cells (112). In germ-free mice that developed metastatic melanoma, CTLA-4 therapy had insufficient antitumor effects due to limited effector CD4+ T cells and tumor-infiltrating lymphocytes, but oral administration of Bacteroides and Burkholderia species improved efficacy of the CTLA-4 therapy with better antitumor effects (121). Similarly, oral gavage of *Bifidobacterium* in mice with B16 melanomas that were treated with PD-1 and PD-L1 was correlated with enhanced DC maturation and their activation of CD8+ T cells, resulting in increased responsiveness to these therapies (122). It is of note, however, that none of the tumors produced by these mice had bacteria found within the tumor tissue. However, in mice with colorectal cancer treated with immune checkpoint blockade therapies (including CTLA-4 and anti-PD-L1), *B. pseudolongum* was isolated from the tumor. This bacterium was identified as essential in upregulating DC-dependent T cell circuitry, thereby boosting the efficacy of the immunotherapies (123). Germ-free or antibiotic-treated mice with MCA-205 tumor cells that were orally gavaged with fecal microbes isolated from immunotherapeutic responders of NSCLC patients had an increase in both CTLA-4 and PD-1 therapy efficacy, whereas oral gavages from non-responders did not improve therapeutic success in these mice (124). These results were also congruent with data from clinical trials, as it was found that melanoma patients who were better responders to PD-1 immunotherapy had greater microbial diversity. These patients specifically had a higher abundance of *Ruminococcaceae* and *Faecalibacterium*, which increased T cell activation by antigen-presenting cells such as DCs (125). Similarly, amongst 42 metastatic melanoma patients, a selection of 8 species of bacteria, including *Enterococcus faecium*, *Collinsella aerofaciens*, *Bifidobacterium adolescentis*, *Klebsiella pneumoniae*, *Veillonella parvula*, *Parabacteroides merdae*, *Lactobacillus* sp., and *Bifidobacterium longum*, were identified in the guts of patients considered responders versus non-responders to PD-L1 inhibitors. These bacteria were associated with Batf3-lineage DCs along with increased activation of T cells (126). These findings were paralleled in a Japanese cohort of patients with advanced NSCLC, in which fecal samples indicated that better responders to immune checkpoint inhibitors (ICI) had a higher abundance of *Lactobacillus* and *Clostridium* compared to non-responders. This was correlated with a longer period prior to failure compared to those who had a lower abundance (127). Supporting all of the previously mentioned studies, it was found that, in advanced NSCLC patients that received antibiotic treatment, there was an increased dominance of *Akkermansia* species in the gut microbiome that was correlated with resistance to PD-1 and PD-L1 therapies (128). Altogether, these findings suggest that the microbiota are tightly correlated with response to therapies, and as such, they stress the significance of exploring changes to the microbiota local to the area of the tumor. Especially in the context of lung cancer, it becomes significant to investigate how these alterations to the microenvironment affect the distinct ecosystem and immunity that exists within the lung.

## 5 Discussion

The relatively recent revelation that the respiratory system is not sterile, but rather home to its own collection of commensal microorganisms, raises many questions regarding their effects on the pulmonary immune system, diseases of the lung, and beyond. This article focuses on the interactions of the microbiota, immune system, and cancers of the lung. Lung cancer comprises a diverse set of pathologies that strongly influence, and are influenced by, the pulmonary niche. A great body of work has described the relationship between lung cancers and pulmonary immunity, and as sampling and sequencing methodologies improve, it will be important to continue to integrate the role of commensal microbiota in this system. This article highlights current findings in this area including how microbes differentially shape the pulmonary immune response and inflammatory environment, and conversely how cancer can shift the lung microbiota through metabolic and immunogenic effects. While progress has been made in defining these areas, studying the lung microbiome presents significant challenges due to the low biomass and dynamic nature of lung microbial communities within and across individuals. This limits the ability to identify microbial “biomarkers” of normal and cancerous lungs. However, it is known that the pulmonary immune system recognizes and responds to local dysbiosis, and that shifts in microbial composition occur during lung cancer progression. The exact mechanisms of these responses, however, need to be worked out in more detail.

One of the biggest challenges in assigning functions to lung microbiota in the context of lung cancer is the overwhelming influence of the gut, which comprises a larger and more stable community of microbiota. Dysbiosis of gut microbiota through disease, genetic factors, diet, and antibiotic usage influences the homeostasis of other systems including lung immunity (129). For instance, lower diversity in the gut microbiota is correlated with early childhood asthma and allergies. Dysbiosis has been linked to a poor response to respiratory infections, likely due to the gut’s role in regulating systemic inflammatory responses. Furthermore, the oral administration of probiotics have been found to attenuate allergic and asthmatic responses in a T-regulatory cell-specific manner (93, 130, 131). In the context of cancer, gut microbiota biomarkers can differentiate between healthy and pre-cancerous lungs. Gut microbial composition is also altered during lung cancer and is predictive of early-stage tumorigenesis, with these changes being linked to shifts in systemic immune signaling (129). However, the mechanisms of the gut-lung axis in lung cancer disease outcome remain unclear. It is necessary to understand how microbial changes to the lung and gut act in parallel to affect lung health, and furthermore to describe the dynamic network of local and systemic immunity that fortify these connections.

Beyond the gut, the lung environment is also influenced by a direct line of communication with the oral cavity. For instance, in humans, transcriptional analysis of airway brushing samples revealed upregulation of ERK and PI3K pathways in the lower airways of lung cancer patients. This transcriptional program was associated with the increased presence of certain oral microbiota taxa including *Streptococcus* and *Veillonella* spp., as measured by 16S sequencing (36, 74). The influence of oral microbiota likely contributes to the dynamic nature of lung microbial communities and presents challenges in discerning specific effects of the lung resident microbiota.

The interaction between extrapulmonary compartments and the lung is not unidirectional. Given the high level of lung vascularization, lung microbiota effects are likely not locally restricted. Just as the gut microbiota influences the biology of distal organs, the lung microbiota may impact extrapulmonary health, as demonstrated in the recent report by Hosang et al. linking the severity of brain autoimmunity to the presence or absence of lung microbiota (132). The lung is also highly innervated, with the nervous system controlling breathing through regulation of airway constriction and dilation. The nervous system is also integrated with lung defense through regulation of cough and inflammation. Neuro-immune crosstalk has been shown to shape the inflammatory response in models of airway allergy and lung bacterial infection (133–135). Recently, in the context of the gut, sensory nerves were shown to mediate tissue integrity through microbiota interactions, suggesting similar systems may be in place in the lung (136). Altogether, this indicates that the nervous system represents another branch to consider in the interaction between the lung microbiota, cancer, and immunity.

In sum, many questions remain regarding the interplay between three complex systems: the lung microbiota, the pulmonary immune response, and the lung cancer microenvironment (**Figure 1**). Technical limitations in sample collection and sequencing methods have slowed the initial pace of discovery, but improvements and standardizations to microbial extraction methods in the lung will greatly improve our understanding of the lung microbiota and how it interacts with the pulmonary space. Subtleties of lung microbiota compositional shifts during lung cancer progression must be studied with greater scrutiny. Many documented interactions between microbes and the immune system are species and strain specific, and as such, improved methods to increase the granularity of species identification are needed to glean more information about their role in lung inflammation and cancer development. Describing microbial functions in the lung at different stages of tumor development, along with their effects on local and systemic immunity, will add insight to understanding cancer progression and the therapeutic efficacy of cancer treatments. Another open area of research is the lung microbiota’s role in nutrient consumption and metabolism; much of our current understanding comes from the gut but understanding these behaviors in lung- and lung tumor-associated microbiota will be informative. While the current gaps in the field may be daunting, they also promise relevant and exciting research opportunities, and most importantly, extend the possibility of improved patient outcomes.

## Author contributions

All authors collaboratively designed the conceptual outline of the review and contributed to all sections. MH integrated the sections that were written separately. All authors contributed to the article and approved the submitted version.
