# Supplementary material for: The lung, the niche, and the microbe: Exploring the lung microbiome in cancer and immunity
Source: Front Immunol. 2023 Jan 17;13:1094110. doi: 10.3389/fimmu.2022.1094110 (PMC9888758; doi:10.3389/fimmu.2022.1094110)
Supplement: Supplementary file 1 [file DataSheet_1.docx]

**Description of Current Tools for Microbiome Analysis**

Because the lung has low microbial biomass, sampling the lung can be difficult and lead to inconsistencies across studies.

16s rRNA sequencing

- Primary method for sequencing analysis in the lung
- Relies on ribosomal RNA
- Less sensitive to host contamination
- Bioinformatic skill required: low
- Gap in Lung Microbiome Research: Consistent protocols across studies

Whole shotgun sequencing

- Greater specificity by sequencing all fragments of genomic
- Allows strain-level interpretation of bacteria, but also fungi and viruses
- Requires low host contamination
- Bioinformatic skill required: intermediate to advanced level bioinformatic skills
- Relies on a well-developed database
- Gap in Lung Microbiome Research: Protocol that has low host contamination

Metatranscriptomics

- Use microbial RNA extracts to derive gene expression from the microbiome
- Describes activity, metabolism, and function of bacteria
- Requires sufficient RNA input for success (Shakya, Lo and Chain, 2019)
- Not well-defined for lung microbiome

Metabolomics

- Mass-spectrometry analysis of metabolites that may be actively utilized or materialized by microbes.
- Not well-defined for lung microbiome

Metaproteomics

- protein and peptides are extracted from a microbial community and are studied to better understand the overall protein landscape in a given environment (Carney *et al.*, 2020)
- Not well-defined for lung microbiome

Culturomics

- Studies conditions and nutrients best promote the overall microbial composition and conditions (Lagier *et al.*, 2018)
- Not well-defined for lung microbiome

These latter four forms of microbial study have not been well-described in the lung, indicating a necessity for a standardized method for extracting and analyzing the lung microbiome and the urgency to develop its research methods.
